# Effective in silico prediction of new oxazolidinone antibiotics: force field simulations of the antibiotic–ribosome complex supervised by experiment and electronic structure methods

**DOI:** 10.3762/bjoc.12.45

**Published:** 2016-03-04

**Authors:** Jörg Grunenberg, Giuseppe Licari

**Affiliations:** 1 Institut für Organische Chemie, Hagenring30, TU-Braunschweig, 38106 Braunschweig, Germany; 2 Physical Chemistry Department, Sciences II, University of Geneva , 30, Quai Ernest Ansermet, CH-1211 Geneva 4, Switzerland

**Keywords:** compliance constants, computational chemistry, drug design, molecular recognition, relaxed force constants

## Abstract

We propose several new and promising antibacterial agents for the treatment of serious Gram-positive infections. Our predictions rely on force field simulations, supervised by first principle calculations and available experimental data. Different force fields were tested in order to reproduce linezolid's conformational space in terms of a) the isolated and b) the ribosomal bound state. In a first step, an all-atom model of the bacterial ribosome consisting of nearly 1600 atoms was constructed and evaluated. The conformational space of 30 different ribosomal/oxazolidinone complexes was scanned by stochastic methods, followed by an evaluation of their enthalpic penalties or rewards and the mechanical strengths of the relevant hydrogen bonds (relaxed force constants; compliance constants). The protocol was able to reproduce the experimentally known enantioselectivity favoring the *S*-enantiomer. In a second step, the experimentally known MIC values of eight linezolid analogues were used in order to crosscheck the robustness of our model. In a final step, this benchmarking led to the prediction of several new and promising lead compounds. Synthesis and biological evaluation of the new compounds are on the way.

## Introduction

**Figure 1:**
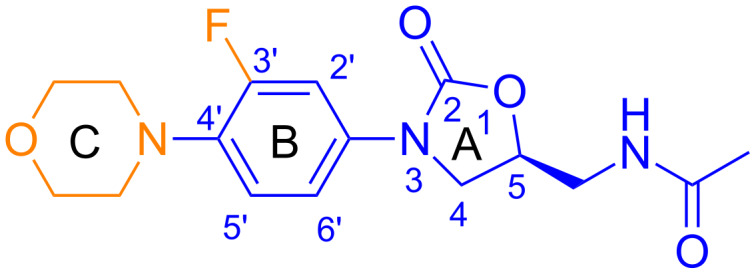
Two-dimensional structure and nomenclature of the oxazolidinone linezolid. The different rings are highlighted with capital letters A, B and C. The acetamidic substituent is denominated as C5 side chain. The pharmacophore portion is colored in blue. Only the *S*-enantiomer is active.

Antibiotic resistance is one of the major health problems in modern societies, causing millions of deaths per year [1–3]. Although Alexander Fleming recognized the importance of the resistance phenomena as early as 1940 [4], in the 1970s the problem of bacterial infection seemed to be solved, because a wide range of potent antibiotics were available. During the last two decades the situation nevertheless changed dramatically: the logarithmic rise in the prevalence of penicillin-resistant pneumococci, for example, led to the description of the situation today as an “Antibiotic Armageddon” [5]. In a recent WHO report it is concluded that “the problem is so serious that it threatens the achievements of modern medicine. A post-antibiotic era, in which common infections and minor injuries can kill, is a very real possibility for the 21st century” [6]. The need for a fast but effective, structure-based strategy for the development of new antibiotics is therefore more than evident [7]. From an experimental point of view, during the last years the amount of structural data describing the bacterial ribosome accumulated. In general, both ribosomal subunits can be targets of several natural or synthetic products, and in most cases, the specific binding sites are within the 16S-rRNA (30S subunit) or 23S-rRNA (50S subunit) nucleotide chains. The nucleotide skeleton of the ribosome therefore plays a central role for the understanding of the relevant recognition processes [8–9]. Examples of antibiotic drug classes that bind the ribosomal 50S subunit are chloramphenicol, puromycin, anisomycin, streptogramina A, and macrolides [10–11]. Those compounds interact with different sites, from the hydrophobic crevice near the entrance, over the so-called A-site (close to the peptidyl transferase region) to the exit tunnel of the nascent polypeptide during the protein synthesis [12]. As a first member of a completely new class of synthetic, antibacterial agents for the treatment of serious Gram-positive infections, linezolid, was introduced in the markets in the year 2000. While also binding to the 50S subunit, it seems to unfold its inhibiting activity in a unique and early stage [13–14]. The nomenclature of the linezolid structure is shown in Figure 1 along with the pharmacophore portion displayed in blue [15]. While only the *S*-enantiomer seems to be potent, linezolid shows activity against a wide range of Gram-positive bacteria such as vancomycin-resistant Enterococcus (VRE), methicillin-resistant *Staphylococcus aureus* (MRSA) and penicillin-resistant *Streptococcus pneumoniae* (PRSP) [16]. Nevertheless, though oxazolidinones represent one of the few new chemical classes of antibiotics disclosed in the past 40 years, cases of oxazolidinone-resistant strains have been already reported [17–19], underscoring again the urgent demand for new linezolid analogues to overcome the growth of bacterial resistance.

To date, only two crystal structures of linezolid bound to its ribosomal target are available: a) linezolid bound to the 50S subunit of *Deinococcus radiodurans* (DR) with a resolution of 3.5 Å [20] and linezolid bound to 50S of *Haloarcula marismortui* (HM) (resolution: 2.7 Å) [12]. As a starting structure for our in silico study, we focused on the latter one (PDB code: 3CPW). This crystal structure was determined in the presence of CCA-*N*-acetylphenylalanine (CCA-N-Phe), an analogue of the portion of aminoacyl and peptidyl tRNAs interacting most strongly with the 50S subunit. It was recognized very early – in fact, even before any crystallographic data were available – that oxazolidinones in general inhibit the bacterial protein synthesis at a very early stage [21–22]. They might impart their inhibitory effect binding the 50S A-site in competition with incoming aminoacyl-tRNA substrates inducing a nonproductive conformation of the PTC (ribosomal peptidyltransferase center). In this context, it is important to note that indeed most linezolid resistance mutations cluster around the PTC, which is exactly the site of peptide bond formation. In addition, the binding pocket for the oxazolidinone ring is characterized by universally conserved 23S-rRNA nucleotides, leading to very similar ligand-bound conformations in different bacterial ribosomes [23].

From a theoretical point of view, the simulation of recognition processes employing nucleotide-based receptors is still hampered today. Difficulties arise because of several issues: Due to the highly charged character of RNA molecules, the electrostatic contributions are quite large. Small, pair-wise errors might sum up to an erratic description of the total energy [24]. Second, the relevant RNA/drug complexes show a pronounced flexibility. It might therefore be misleading to focus on the solid state structures of available nucleotide/guest complexes alone [25]. A third obstacle is the intrinsic deficiency of empirical force fields especially for DNA/RNA structures (“force-field polymorphism”). A thorough force field method evaluation is therefore a prerequisite for any meaningful in silico study, especially of processes that involve molecular recognition by DNA/RNA hosts [26].

Though there are many success stories in the literature, it is not all it's cracked up to be in the euphoric 1990s, when computer-based drug design was one of the big scientific promises. New drugs are nowadays produced at the same rate as they were 60 years ago [27], notwithstanding enormous investments and undeniable achievements both, in computer soft- and hardware. Modern computational drug design strategies range from A) virtual screenings [28–30] of large molecular databases to B) sophisticated simulations of the state equations [31–32]. Nevertheless, both strategies have their own advantages and disadvantages when it comes to the reliable prediction of new drug candidates. While fast virtual screening methods, based on scoring functions, are able to tread hundred thousands of candidates in a highly approximate (and sometimes erratic) fashion, alchemical-free energy perturbation (FEP) calculations are also error prone. The predictive power of those FEPs depends on many adjustable parameters, even if the underlying force field description itself is perfect.

Our proposed strategy therefore relies on three pivotal points: 1) The systematic evaluation of the underlying empirical force field, 2) a heuristic approach in order to choose potential drug candidates and 3) an effective scan of the relevant receptor/candidate energy surface.

## Overall Strategy

Because the quest for new and effective oxazolidinone binders is a typical lead optimization problem, our strategy in order to predict new linezolid analogs is therefore settled just in middle between these two extremes: a combination of computational power and chemical heuristic [33]. In order to identify a few, but promising, molecular candidates out of a manageable subgroup of potential candidates, we applied a robust, but fast force field method, focusing on the endpoints of the recognition path. These endpoints were nevertheless characterized by elaborate scans of the relevant conformational hyper surface. Further, since in cases of high chemical similarity, our assumption is that the enthalpy governs the recognition process anyway [34–35]. We thus further assumed a constant entropic contribution to the Gibbs–Helmholtz equation for all candidates, focusing on the realistic description of all enthalpic contributions. Since the whole ribosomal system is by far too complex for any systematic all atom in silico study, we – in a first step – constructed a truncated model of the ribosome, followed by a thorough conformational search of Linezolid and its analogues complexed inside the model ribosomal active site. Sampling the lowest energy wells in a 40 kJ/mol energy window the lowest enthalpy minimum was used to assess the relevant terms in Equation 1 below. Since the linezolid complex represents the zero point of our relative binding energy scale, a negative ΔΔ*Eb* value denotes a more favourable interaction of the linezolid analogue with the ribosome.

The PDB structure 3CPW was used as it is, including the CCA-N-Phe moiety. Conformational searches were done applying the stochastic Monte Carlo (MC) method. All scans were carried out for a) the isolated guests and b) the guests bound into a cavity model (see below) based on the *Haloarcula marismortui* crystal structure. An implicit solvent model was used throughout. Relative enthalpic binding energies were calculated according to Equation 1.

[1]
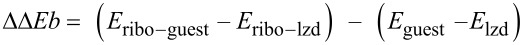


where ΔΔ*Eb* represents the binding enthalpy relative to linezolid considered as the zero point of our scale. Since in our case, the receptor is represented by the same ribosomal model derived from PCB structure 3CPW the relevant terms are the following: the enthalpy of 1) the complex between the ribosome and particular linezolid analog (*E*_ribo-guest_), 2) the complex between the ribosome and linezolid (*E*_ribo-lzd_), 3) the solvated linezolid analog (*E*_guest_) and finally 4) the solvated linezolid (*E*_lzd_).

### Force field evaluation: bioactive conformation of linezolid

**Figure 2:**
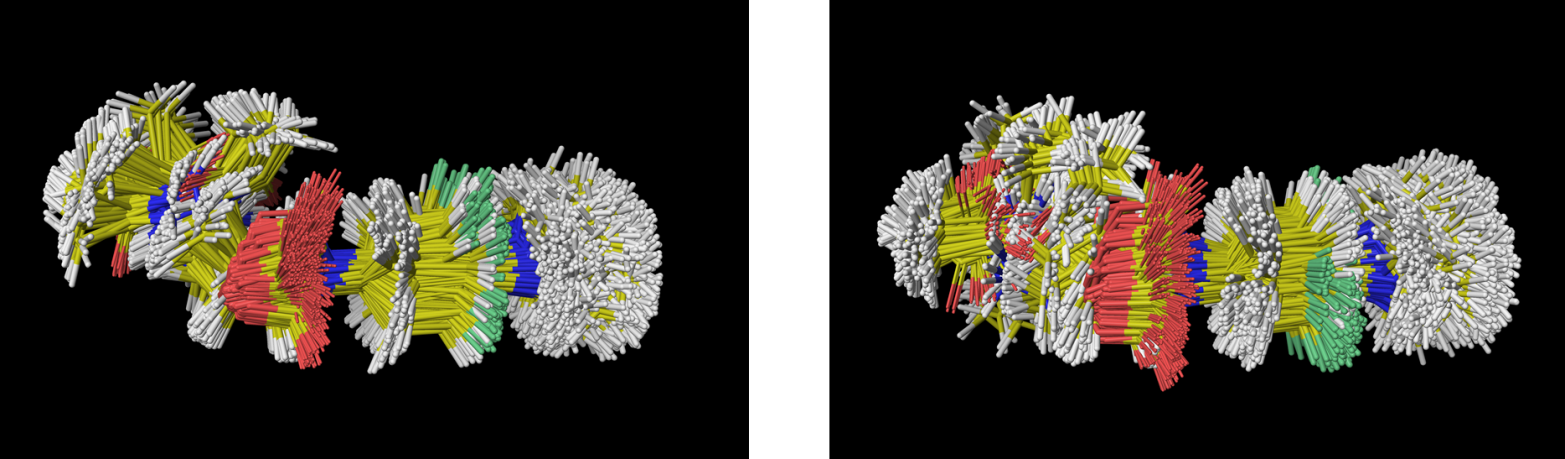
Superposition of all low energy minima of linezolid applying AMBER (left) and OPLS-AA (right) force field.

**Figure 3:**
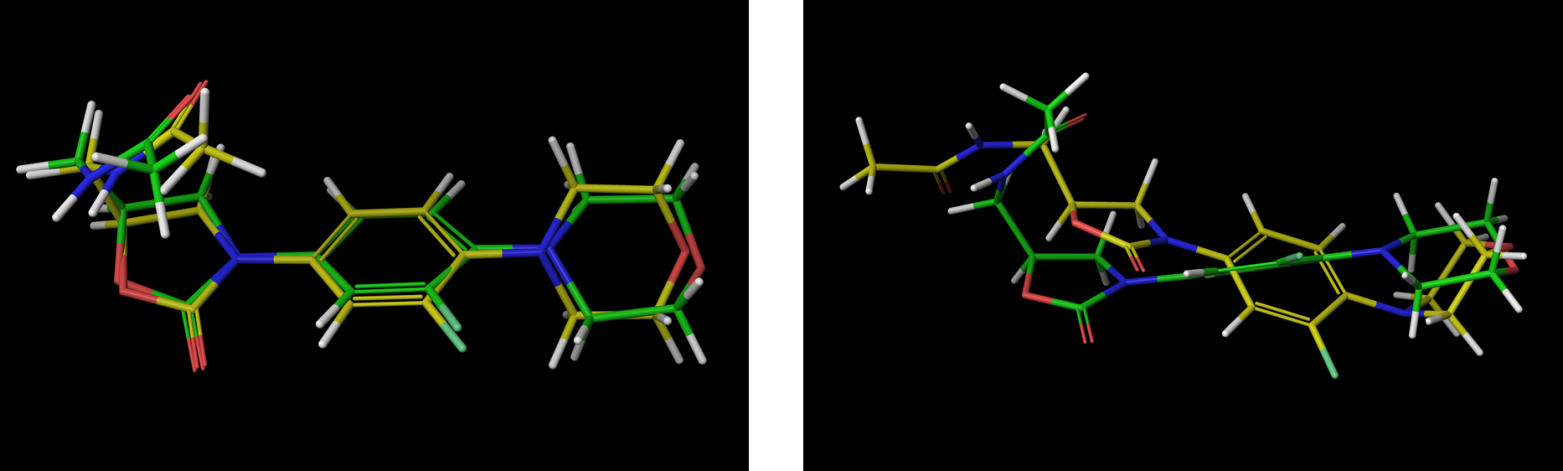
Superposition of the found global linezolid gas phase minimum (AMBER on the left and OPLS-AA on the right) with the linezolid bioactive conformation (green). The OPLS-AA force field reproduces the conformation linezolid adopts in the solid state [38] or complexed by a transporter protein [39].

Since the description of any molecular recognition process relies, first and foremost, on an authoritative reproduction of potential energy surface of the studied system, we started our analysis with a force field evaluation comparing the AMBER and OPLS-AA implementations (see Supporting Information File 1 for details) as a test case. While these two force fields are quite similar concerning the overall structure of the functional form, subtle differences in individual atomic parameters might nevertheless accumulate and lead to an erratic description of the overall energy [24]. We therefore run extensive Monte Carlo (MC) conformational scans for the isolated linezolid in order to find out if the particular potential function is able to reproduce a) the unbound and b) the bioactive structure of linezolid. Although the gas phase or solvent minimum structure of unbound linezolid is not known experimentally, we assume that it is very similar to the bioactive conformation [36–37]. Our strategy is based on the following assumption: in order to avoid an enthalpic penalty, the conformer of a drug-like molecule in the unbound state has to be similar to the bioactive conformational state. The result of our MC scans is shown in Figure 2. An overall number of ca. 450 low energy (40kJ/mol window) conformers were sampled, both for the AMBER and the OPLS-AA force field, respectively. Both methods are capable to describe the overall flexibility of linezolid, particularly the orientation of the acetamidic side chain. In Figure 3 the lowest minima were superimposed and compared to the bioactive linezolid conformation. While the AMBER global minimum is characterized by a small RMDS of 0.73 (Figure 3), the OPLS-AA scan ended up with a global minimum, that differs dramatically from the bioactive conformation (RMSD of 2.3).

The side chain of the global OPLS-AA minimum is oriented pseudo equatorial with respect to the plane of the molecule, instead of an overall bent bioactive conformation. The OPLS-AA force field seems to underrate the attractive long-range interactions between the amidic group and the oxazolidinone ring.

**Figure 4:**
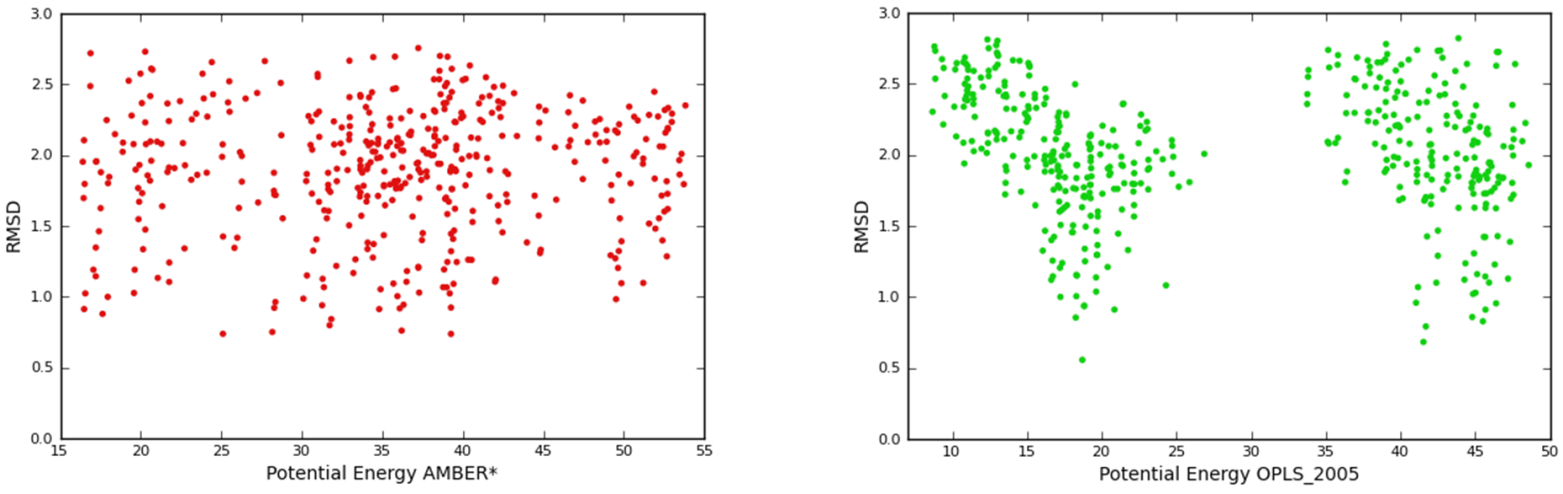
RMSD/Potential energy plots for linezolid in the gas phase. The OPLS-AA plot is characterized by an absence of conformers roughly between 25–34 kJ/mol.

A second important difference between the two force fields is shown in Figure 4: Our RMSD plot for all sampled conformers shows that the AMBER force field produces a homogeneous distribution of the low energy conformations, while the OPLS-AA low-RMSD structures are clustered in confined areas with a 10 kJ/mol wide conformer gap between 25–35 kJ/mol. We finally evaluated our force fields results comparing them with first principle DFT calculations. For both force fields the, 10 lowest conformers were used as a starting point for our DFT optimization in order to check if the same energetic order was achieved. The OPLS-AA force field produces an artifact: a global minimum, which is higher by ca. 20 kJ/mol in comparison with DFT. Any method, which is unable to describe the conformational space of the isolated guest, will of course also predict erratic results during the simulation of relevant recognition process. We therefore decided to use the AMBER force field throughout this work. The term *E*_lzd_ in Equation 1, therefore correspondents to our AMBER results throughout.

### Ribosomal model building

**Figure 5:**
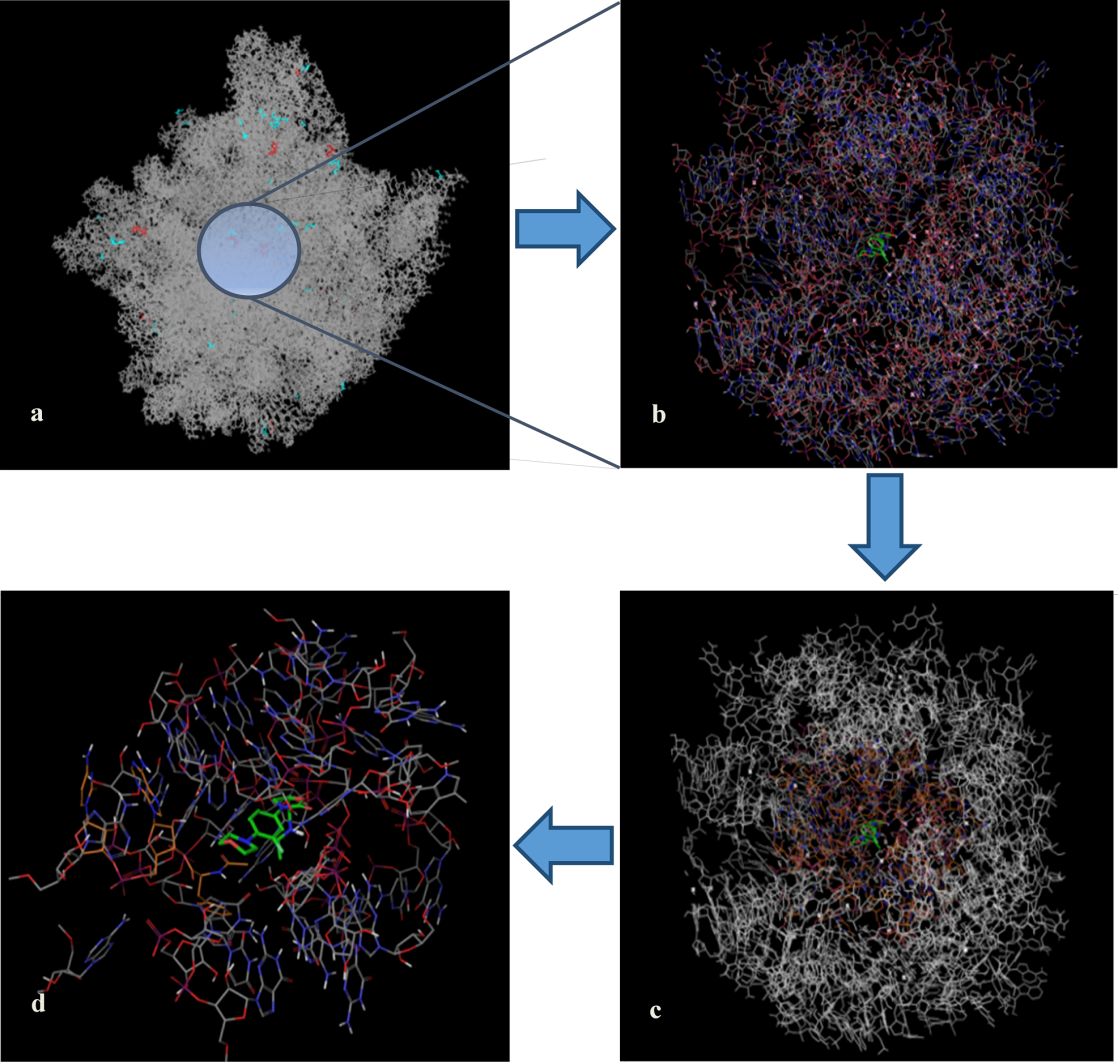
Model building process. a) linezolid bound to the 50S subunit from *Haloarcula marismortui*, code 3CPW, ca. 100 000 atoms; b) 30 Å radius mother-shell pulled from the crystal structure, linezolid in green; c) optimizing the mother-shell; d) 10 Å radius working-shell used for the conformational searches throughout the paper.

Four different interaction shells of decreasing complexity were constructed successively (Figure 5). The first shell was constructed from the crystal structure of linezolid bound to 50S of *Haloarcula marismortui* (I, Figure 5a). All the atoms within 30 Å distance from bound linezolid were selected and the relevant residues were expanded. The missing hydrogen atoms were added, water molecules beyond 5 Å from hetero groups were deleted (Figure 5b). Crystallographic water molecules were treated explicitly if they were part of the hydrogen bond network. This working shell was the starting point for the construction of all complexes models used during our MC scans. It includes linezolid, the CCA-N-Phe unit, nucleotides belonging to the r-RNA backbone, a few amino acids, water molecules and cations. In a second step, models of different size were cut out from this working shell in order to find the smallest model still including all relevant moieties affected by linezolid inside the active site (working shell I). The resulting ribosomal model still characterized by 1) the reproduction of linezolid in its bioactive conformation and 2) the existence of all non-covalent interactions known from the crystal structure, was finally the starting point for our conformational scan. All Mg^2+^ and Sr^2+^ ions of the mother-shell were replaced by Ca^2+^, the partially cut residues were completed. In order to relax the maximal forces, this very mother-shell was optimized applying the AMBER force field (gradient of 2.0) freezing only the outermost atoms. That means, within a shell of 12 Å (measured from linezolid's center of mass) all atoms were optimized (Figure 5c). From this optimized shell a second one (working shell II) was extracted, selecting all atoms within 10 Å from linezolid, expanding the partially cut residues again. The outer phosphate groups bound to 5’ position of ribose, affected during this cutting process, were replaced by methoxy groups. An overall number of 10 Na^+^ cations were added randomly outside the 10 Å shell in order to counteract in part the highly negative charged phosphate back bone. This newly added ion shell was minimized again under a gradient of 0.3, fixing the position of all the atoms. The optimized structure (Figure 5d) represents our final working-shell III consisting of 1623 atoms, 131 residues and all in all 97 molecules.

### Ribosomal stereoselectivity: the (*S*)-linezolid complex

Our described model of the complex between the ribosome and the linezolid guest was used as a starting point for the series of our Monte Carlo scans. In order to limit the computing time, only the guest’s internal and external (translational/rotational) degrees of freedom were included as scanning variables at this step. Again, the flexibility was retained in part: each Monte Carlo step was followed by a partial optimization of the complex, defining a substructure of freely moving atoms within 5 Å from linezolid (557 atoms). The remaining atoms were fixed (1022 atoms). In the following, this very definition of the adaptive sub-shell has been used for all our ribo/guest complexes. Because the energy of this low minimum represents the zero point of our relative affinity scale, we intensified our scan at this point in order to make sure that we will not produce false positive hits for the linezolid analogues. An initial phase with 5000 steps produced a low energy conformer very similar to the conformation of linezolid in the crystal structure. After another 5000 steps (starting from the previous minimum), a new lowest minimum very similar to the solid state conformation was detected lying 31.8 kJ/mol below the previous one. Another 5000 steps, now 15000 in total, nevertheless did not lead to a lower minimum. Hence, 10000 MC steps were chosen as total number of steps for the complexes’ conformational searches. Of course, this does not fully guaranty that we really found the “absolute” global minimum but seems to be a reliable compromise to us in order to keep the protocol as practical as possible.

**Figure 6:**
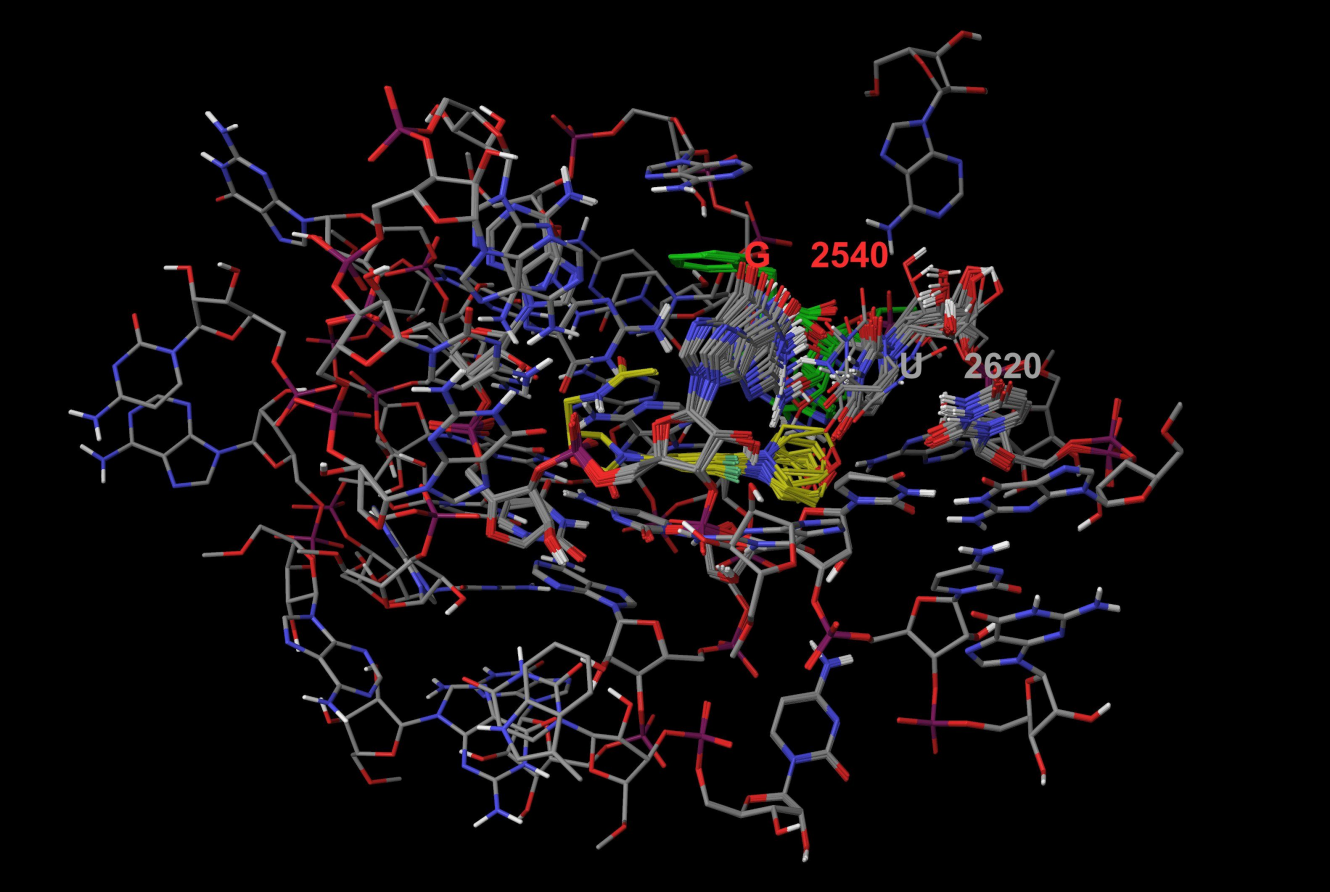
Superposition of all 43 minima of the ribosome–linezolid complex. Linezolid is shown in yellow and CCA-N-Phe in green. The nucleotide nomenclature is the same as the one reported in the crystal structure original paper [12]. Note the bent conformation of linezolid in its bioactive state and the flexibility of the morpholine ring.

A total number of 43 individual conformers, belonging to individual energy wells were collected within a 40 kJ/mol window (Figure 6). The following points can be concluded from our computations:

1) The pharmacophore portion of linezolid is structurally preserved. 2) The H-bond between the linezolid side chain and G2540 phosphate group (*Haloarcula marismortui* numbering used throughout) is present in all conformers. 3) The equilibrium H-bond length of 1.70 Å and the calculated relaxed force constant [40] of 0.31 N/cm point to a strong interaction [41], which is decisive for the recognition process.

According to experimental results [11], the morpholine ring is not directly involved in the molecular recognition process. Our simulation could reproduce this high flexibility of the morpholine moiety, too (see Figure 6). Turning to the conformational changes in the receptor, they seem to be localized at the nucleobases G2540 and U2620 as well at the CCA-N-Phe unit. The flexibility of the U2620 (U2585 in *Escherichia coli*) moiety has already been discussed elsewhere [20]. Nevertheless, due to our simulation, there is no hydrogen bond between this nucleobase and the morpholine ring. The absence of a second hydrogen bond in our simulations (which is indeed observed for linezolid bound to the *Deinococcus radiodurans* 50S subunit) can be rationalized: the binding of linezolid into the ribosome stabilizes the position of U2620 (U2585 in Escherichia coli) in a slightly different (but decisive) orientation depending on the occupation of the A and P-site with tRNA ligands. Comparing our simulated bioactive conformation of linezolid with that from linezolid bound to *Haloarcula marismortui* (code 3CPW), we find a value of 0.70 RMSD, again pointing to the quality of our ribosomal model. Interestingly the nucleobase G2540 (U2505 in *Escherichia coli*) undergoes a profound conformational change during the Monte Carlo scan. While in the solid state structure from *Haloarcula marismortui* this nucleobase is oriented away from linezolid, due to our simulation the nucleobase approaches linezolid interacting through a new H-bond (relaxed force constant: of 0.23 N/cm) between the NH_2_ group and the morpholine nitrogen. Though not observable in the solid state, this movement increases the binding affinity of linezolid under biological conditions. Due to our simulation this low energy minimum is populated 83% of the time at 310 K, dominating the recognition process.

We repeated our protocol calculating the various conformations of linezolid inside the ribosome, applying the OPLS-AA force field. Indeed both, the lowest energy minimum and the 10 following conformations are characterized by a side chain, which is oriented pseudoequatorial with respect to the plane of the molecule, instead of a bent conformation known from the bioactive solid-state conformation (see Supporting Information File 1, Figure S1). The OPLS-AA force field seems to underrate the attractive long-range interactions between the amidic group and the oxazolidinone ring also in the bound state. We would like to emphasize that this inferiority of the OPLS-AA force field is not an overall phenomenon. It rather underlines the importance of a thorough force field evaluation for each survey.

### Ribosomal stereoselectivity: the (*R*)-linezolid complex

**Figure 7:**
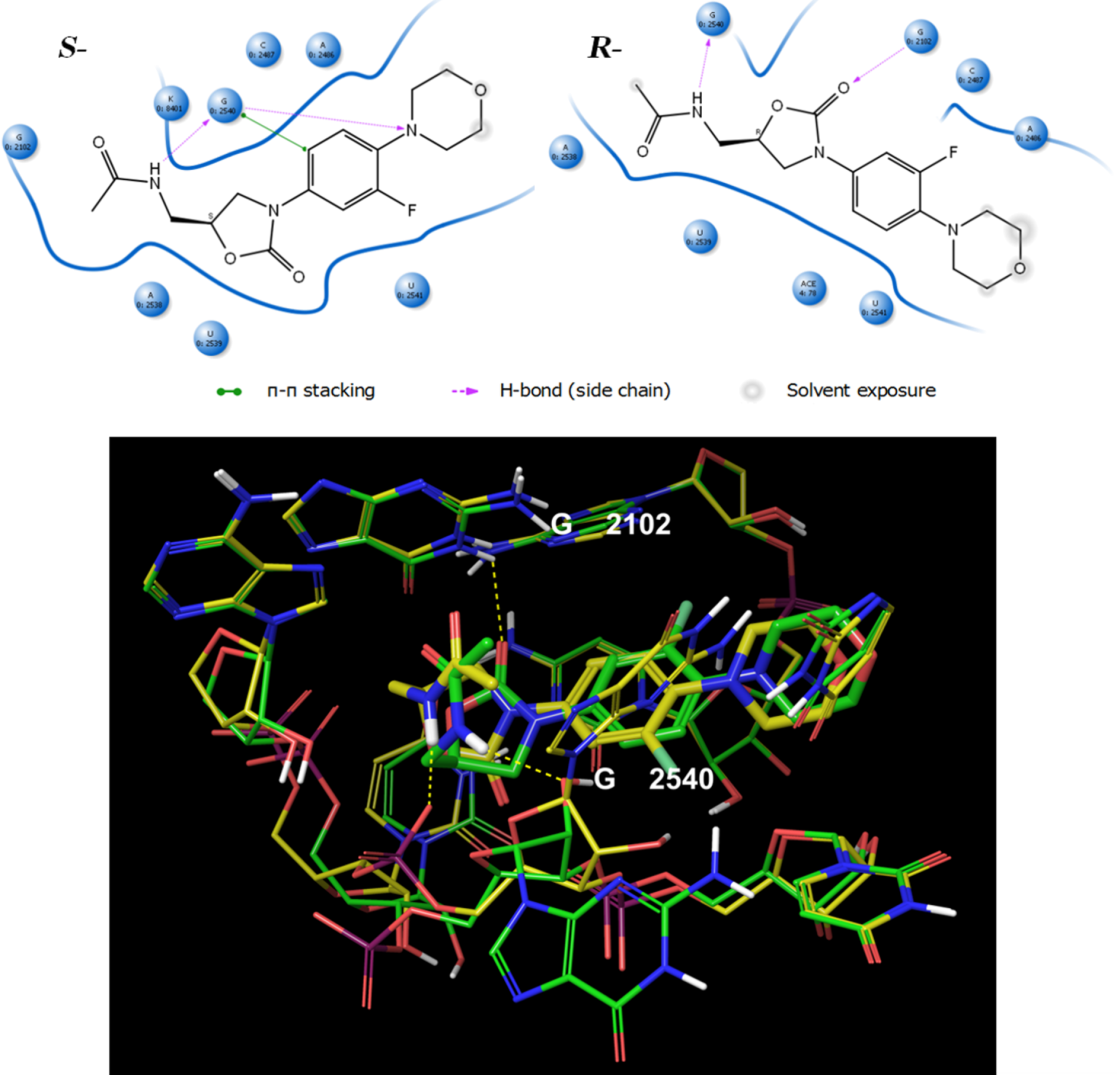
Comparison between ribo/S-lzd and ribo/R-lzd (quasi) global minima. On top, a 2D interaction diagram is displayed. A zoom of the found minima superimposed each other that illustrate the conformation and interactions made by S-lzd (yellow) and R-lzd (green).

As an acid test for the robustness of our model we tried to reproduce the enantioselectivity (it is well known that (*S*)-linezolid is the only active enantiomer) of the recognition process [16,42]. Again, the working-shell was used for this purpose as starting structure. The chirality of linezolid was inverted in place inside the receptor. (*R*)-Linezolid was rotated by 180° around an imaginary axis passing along the ring planes. This caused the side chain to occupy approximately the same position as in the “bent” bioactive conformation of the (*S*)-linezolid. The Monte Carlo scan was started running with the same settings as for the ribo/S-lzd. After 10000 steps the number of sampled energy well was twice as high (88 conformers). Our (quasi) global minimum for the ribo/R-lzd complex, occupied 51% of the time at 310 K. The following points are striking: 1) in terms of the enthalpic contribution, the ribo/R-lzd complex seems to be dramatically less stable in comparison with the ribo/L-lzd system. Our calculated enthalpy is 24.1 kJ/mol higher than lowest minimum of the ribo/S-lzd system. 2) Due to our simulation the experimentally known enantioselectivity can be rationalized on an atomistic level of resolution: while present in both ribosomal/linezolid complexes (R-lzd and S-lzd adopt a similar conformation with the same spatial orientation), the NH···O=C hydrogen bond in R-lzd shifts from the negative charged G2540 phosphate group to the ribose 2’-hydroxy group (Figure 7). In line with the reduced negative charge of the acceptor, the new interaction is weaker and the bond length is increasing from 1.70 Å to 2.37 Å (relaxed force constant: of 0.18 N/cm). 3) Taking a closer look at the R-lzd complex, there also seems to be a very soft H-bond interaction between the oxazolidinone-ester-type oxygen and the G2102 guanine NH group with a bond length of 2.43 Å and a relaxed force constant of 0.12 N/cm. This observation confirms the ester-type oxygen in the oxazolidinone ring as a hydrogen-bond acceptor. 4) Most important, the hydrogen bond between the G2540 nucleobase and the morpholine ring of linezolid is completely absent in the host/R-lzd complex. The nucleobases are twisted away from the drug leaving the R-lzd NH group free to interact with the G2540 2’-hydroxy moiety.

All in all, while we cannot exclude other bacterial defensive processes, the inactivity of R-lzd indeed seems to be related to a low enthalpic contribution during the recognition by the ribosome, relative to the strong binding *S-*enantiomer.

### Reproduction of experimental MIC values

**Scheme 1:**
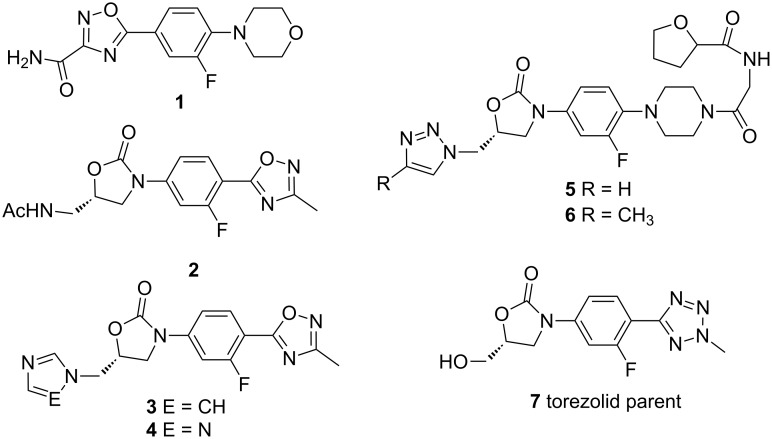
Experimentally characterized linezolid analogues that were used as test cases for our simulation protocol. Compound 7 is the active form of the prodrug torezolid. For references, see the text.

As a next step in our supervised modeling strategy, we analyzed seven experimentally characterized linezolid analogues (see Scheme 1) applying our simulation protocol, in order to test the robustness of our ribosomal model. A direct comparison of in silico enthalpic affinities to the ribosome with in vitro MIC values (minimal inhibitory concentration) is, of course, not straightforward. Nevertheless, again applying our semi-heuristic strategy, a rough picture concerning the quality of our protocol can be drawn. Table 1 summarizes our computed relative enthalpic binding affinities of eight linezolid analogues against our ribosomal model compared with their experimentally known antibacterial activities, expressed as MIC values. We come to the following conclusion: while the discrimination between good and bad binders seems to be overrated by our computer model, all candidates with a high experimental MIC value are correctly described as bad binders. This result is especially important in order to exclude false positive results. Compound **1** [43] bearing a reversed amide moiety, does not show any experimentally activity against a variety of bacterial strains, in line with a simulated enthalpic penalty of +8.8 kJ/mol. It has been suggested by Palumbo Piccionello et al., that, though the 1,2,4-oxadiazole ring is isosteric with the oxazolidinone ring and possesses similar hydrogen bond acceptors sites, the lack of biological activity is connected with a prohibited cellular uptake. Despite our model cannot tell anything about the bioavailability, metabolic half-life or side effects, due to our computations, compound **1** would have a lower affinity than linezolid even if it arrives at the ribosomal target. Though **1** contains a NH_2_ group that is able to form a weak H-bond, its amidic character and the lack of a chiral center leads to a side chain orientation parallel to the 1,2,4-oxadiazole ring, changing the overall binding mode in comparison with linezolid, and by this decreasing the overall binding affinity. Our calculated enthalpic penalty for **1** of +8.8 kJ/mol is in line with the observed inactivity (MIC value >256 mg/L). The same holds true for candidate **4**, substituted by a 1,2,4-triazol-1-yl function, with a predicted penalty of +90 kJ/mol and an observed MIC value of >50 mg/L.

**Table 1:** Computed relative binding energies in comparison with MIC values for the experimental compounds tested on the model.

Guest	ΔΔ*E_b_* [kJ/mol]	MIC value^a^

**1**	+8.8	>256
**2**	−15.6	2
**3**	(−3.4)	>50
**4**	+90.0	>50
**5**	−5.2	8
**6**	+7.7	32
**7**	−27.9	0.5
linezolid	0.0	1–2

^a^Minimal Inhibition Concentration values expressed in mg/L for selected methicillin-resistant *Staphylococcus aureus* bacterial strains (MRSA). The reason for the false positive value of compound **3** is not clear. We therefore put the value in parenthesis.

Turning to the linezolid analogues **2** and **3**, the morpholine ring was replaced by a 3-methyl-1,2,4-oxadiazole ring [44]. Whereas **2** maintains the linezolid C5 side chain, compound **3** is substituted by a imidazole-1-yl. With its MIC value of 2 mg/L compound **2** seems to be comparable (or even more effective against the MRSA 433 strain, see lit. [44]) as linezolid, **3** does not show any remarkable activity (MIC value >50 mg/L).

Again, in terms of our semi-heuristic ansatz, this overrating is welcomed in order to exclude false positive results. The experimental activity of compound **2** is almost identical to that of linezolid, while our calculated relative binding energy of −15.6 kJ/mol seems to be lower bound for the prediction of an effective binder. The experimental ineffectiveness of compound **3** (MIC value >50 mg/L) is mirrored by our computed decrease of the enthalpic contribution by more than 12 kJ/mol. Two earlier in silico approaches [44] based on *Deinococcus radioduran*'s 50S ribosomal subunit suggested that the binding mode of **2**, **3** and **4** is very similar to that reported for ribosome–linezolid complex crystal structure. The 1,2,4-oxadiazole ring is able to mimic the role of the morpholinic ring. In addition, our simulations predict that at least in the case of compound **2**, there still is a stabilizing H-bond between the NH group in the side chain and the hydroxy group of the U2539.

Compounds **5** and **6** belong to a family of triazolyl oxazolidinone substitutes, bearing an N-substituted piperazino moiety at the 4 position of the phenyl ring. They contain several additional H-bond donor and acceptor functions at the terminal *N*-glycinyl position [45]. This class of compounds shows moderate to potent antibacterial activity against staphylococcal and enterococcal strains. Though both candidates differ just by a single methyl substituent in 4 position of the triazolyl group, this change nevertheless has a pronounced effect: the biological activity for **5** (MIC value: 8 mg/L) is 4-fold in comparison with the linezolid analog **6** (MIC value: 32 mg/L). Again, our computer model is able to reproduce this relative binding abilities, induced by subtle chemical differences (H vs CH_3_) favoring **5** over **6** by more than 13 kJ/mol. An inspection of our obtained minima reveals that, whereas the flexible terminal *N*-glycinyl chain is capable of making different water mediated H-bonds or with the residues from the active site entrance directly, the remaining pharmacophore part fits perfectly into the binding site. What makes the difference in the relative binding energies between those two compounds seems to be the steric repulsion exerted by the methyl group of **6**. This repulsion causes a twisting of the G2540 nucleobase and hence the rupture of the important H-bond with the morpholine ring in all low-energy conformations.

Finally, due to our computations, the best enthalpic binder should be the linezolid analog **7**, characterized by a predicted enthalpic reward of −28 kJ/mol. Indeed, experimentally this candidate revealed an excellent potency both in vitro and in vivo antibacterial activities [46]. Candidate **7** represents the active form of the prodrug torezolid, the phosphate disodium salt, which was synthetized in order to improve the solubility of its parent drug. Highly active against MRSA, MSSA (methicillin-sensitive *Staphylococcus aureus*), VRE and Hi (*Haemophilus influenzae*) bacterial strains, it is currently undergoing clinical trials. An earlier docking study of **7** by Shaw et al. [47] postulated an increased potency mediated by additional interactions between the ribosomal active site and the pyridine and tetrazole rings from **7**. Particularly, two H-bonds between the sugar backbone of residues A2451 and U2584 (EC numbering) may be the main responsible for the increased potency. Due to this earlier study, the hydroxy functionality in **7** is donating the hydrogen bond superseding linezolid’s acetamide hydrogen donor. Indeed our stochastic conformational scan of the ribosomal complex of candidate **7** revealed exactly those interactions: four low energy conformers, contained within an energy window of 3 kJ/mol, were identified accounting for 79% of the Boltzmann population. Most of the low-energy conformers adopt a linezolid-like binding mode: the hydroxy group is stabilized by a hydrogen bond with the phosphate of G2540 (relaxed force constant: 0.20 N/cm). Furthermore, the pyridine group establishes a second hydrogen bond with the sugar of G2541 (relaxed force constant: 0.21 N/cm), confirming the capability of this substructure to interact with the nearby A-site entry.

Overall, our chosen ribosomal model in combination with the simulation protocol seems to be robust enough to allow a discrimination between “good” and “bad” binders. The general MIC trend of the experimentally characterized candidates is reproduced: bad ribosomal binders, classified by a calculated enthalpic penalty, show high MIC values (candidates **1**, **4** and **6**). Four candidates have been found to possess a negative ΔΔ*E_b_* relatively to linezolid, characterizing those derivatives as good binders. The reason for discrepancy for compound **3** is still under examination. We cannot exclude an experimental error of course, but we are far away from making any proposition in this context.

Without knowing the experimental MIC values in advance, choosing the candidates **2**, **3**, **5** and **7** according to their computed negative enthalpy would have been an excellent selection with respect to further in vitro studies.

### New linezolid analogues

**Scheme 2:**
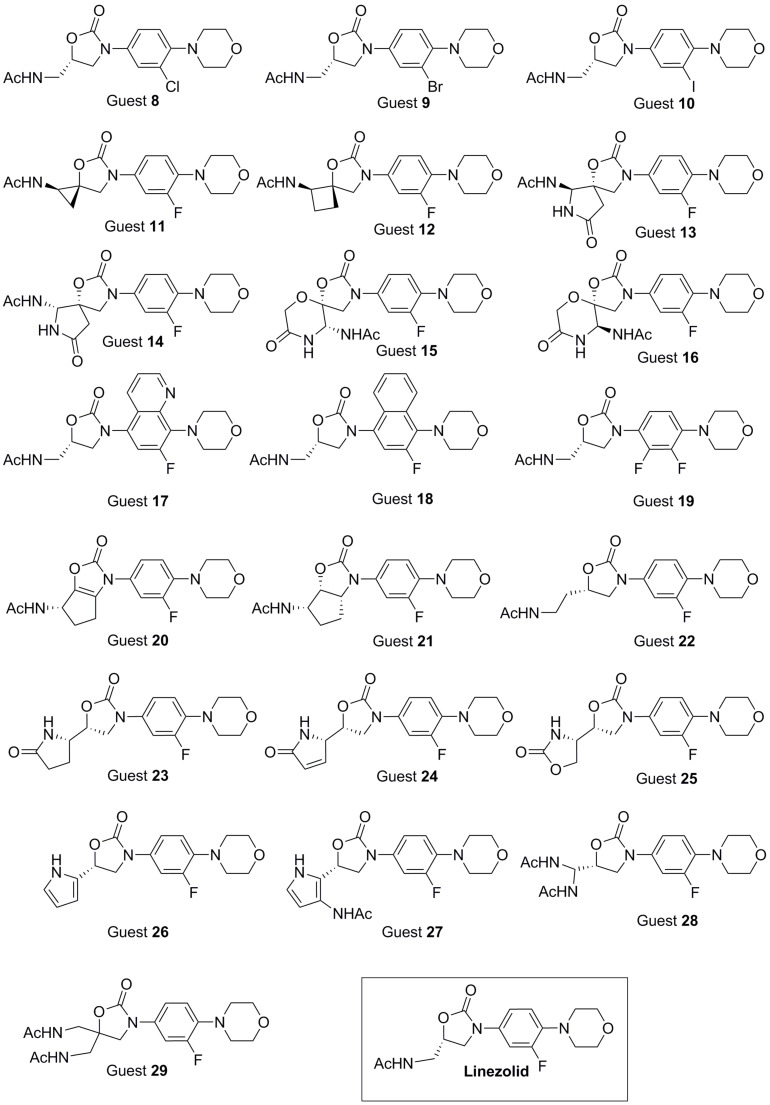
Predicted new linezolid-like candidates.

After this evaluation of both, the applied force field and our ribosomal model, we were finally in the position to predict new linezolid-like candidates. Scheme 2 compiles our candidates for the simulation, which were chosen solely on the basis of chemical intuition. The values of the enthalpic contribution to the ribosome affinity were calculated as described before. Only guests **8** and **9** have been reported earlier, nevertheless in another context [48]. Their biological activities are not known. The rest of our candidates were designed from scratch bearing the same stereochemistry as linezolid, though in some cases the absolute configuration can shift from *S* to *R*. Candidates **13**/**14** and **15**/**16** are diastereomers. Guests **8**, **9**, **10** and **19** were included in our test set in order to probe if different halogen atoms (B ring) influence the affinity. It is known that spirobicyclic compounds have a high affinity towards the ribosomal A-site [49]. We therefore designed candidates **11** to **16** resembling this spirobicyclic scaffold while keeping the CH_3_CONH group as H-bond donor. Additionally, this modification is connected with a higher rigidity of the side chain.

Further B ring modifications were introduced in candidates **17** and **18**. Those candidates might lead to a better stacking interaction between the fluorophenyl moiety and the heteroaromatic crevice (A-site cleft). In addition, a larger aromatic system could lead to a closer contact between the B ring and the nucleobases A2486 and C2487. In order to generate a higher rigidity, two additional guests (**20** and **21**) were included in our set of candidates, connecting the side chain to linezolid’s A ring. In guest **22**, the side chain was extended by an additional CH_2_ unit. Guests **23** to **26** are characterized by further modifications at the side chain, keeping neverthelss the NH group as a hydrogen bond donor. Finally, we added a second CH_3_CONH group to the side chain (candidates **27** and **28**). A further H-bond donor could improve the enthalpic affinity between the drug and the receptor by increasing the chance for a second H-bond between the acetamidic arm and the G2540 phosphate group.

Table 2 summarizes the results for the calculated relative binding energies. Due to our simulation, three among the 20 new linezolid analogues show a higher binding affinity in comparison with linezolid, which is not too bad for a first hit rate. In the following we will discuss nevertheless not only the 15% good binders but all our candidates. Guest **8** shows a negative relative binding energy (−3.7 kJ/mol). Its lowest minimum conformation inside the receptor exactly fits the orientation of complexed linezolid. The small but negative relative binding affinity can be explained by an improved H-bond interaction (relaxed force constant: 0.27 N/cm) between the softer chlorine atom and the hydroxy group in the ribosome 2’-position of G2540.

**Table 2:** Predicted relative binding energies new oxazolidinone derivatives in comparison to linezolid. Negative values refer to compounds with a higher affinity than linezolid.

Guest	ΔΔ*E_b_* [kJ/mol]	Guest	ΔΔ*E_b_* [kJ/mol]

**8**	−3.7	**19**	+42.4
**9**	+83.5	**20**	−12.6
**10**	+40.9	**21**	+56.8
**11**	+21.2	**22**	+40.2
**12**	+68.3	**23**	+9.9
**13**	+25.7	**24**	+25.8
**14**	+30.8	**25**	+1.3
**15**	+76.7	**26**	−7.0
**16**	+58.5	**27**	+39.1
**17**	+45.2	**28**	+36.8
**18**	+39.9		

In contrast, the lowest energy minimum of guest **9**, while also well superimposable to bound linezolid, lacks the H-bond interaction between the G2540 nucleobase and the morpholine ring. The missing hydrogen bond in combination with a large conformational change of the whole guest leads to a severe destabilization of guest **9** (+85.5 kJ/mol) bound to the ribosome. In the case of candidate **10**, the big iodine atom again causes a complete change in the guest recognition mode by the receptor (+40.9 kJ/mol): the H-bond is now located between the NH donor portions and the nucleobase U2539 instead of the G2540 phosphate. The substitution of a second fluorine atom, guest **19**, resulted also in a thermodynamic destabilization by +42.4 kJ/mol. This penalty seems to be the result of additional steric strain: the second fluorine pushes the G2540 ribose ring out of its original position. The H-bond of the morpholine ring is broken. We speculate that this observed high flexibility of the G2540 residue is of pivotal importance for the overall binding process: in fact, if G2540 is interacting with the morpholine ring, the drug is “clamped” in the binding site, because its side chain is stabilized by stacking interactions.

While our model recognize different enantiomers (guest **13** and **16** are stabilized in comparison with guest **14** and **15** by 5.1 kJ/mol and 18.2 kJ/mol, respectively) none of the spiro-bicyclic compounds shows a promising affinity to the bacterial ribosome. The same is true for guests **17** and **18**. The introduction of the new aromatic cycle in the B ring increases the steric hindrance with far-reaching structural consequences: the H-bond to G2540 phosphate is disrupted. Further, an additional CH_2_ group in the side chain (guest **22**) does not lead to any promising affinity (+40.2 kJ/mol). The pivotal hydrogen bond is lost. The two weak mediated H-bonds between the ester-type oxygen in the oxazolidinone ring and a water molecule (2.25 Å) and one between morpholine oxygen and 2’-hydroxy group in CCA-N-Phe adenosine ribose (1.91 Å) are not able to compensate this enthalpic penalty. (For a recent detailed study on the importance or unimportance of mediated hydrogen bonds see reference [25].)

Guests **27** and **28** containing a second side chain show unfavorable overall binding enthalpy, too. The large steric demand introduced with this modification moves both compounds slightly outwards the A-site: the pivotal hydrogen bond with the G2540 phosphate group again is lost. Guest **27** is even deprived of a NH unit by an intramolecular H-bond between the NH of one side chain and the carbonyl of the second. The introduction of a heteroatomic or heteroaromatic 5-membered ring in the side chain led to quite promising candidates: guest **23**, for example, is predicted to have a binding affinity not far from linezolid itself (ΔΔ*E_b_* = +9.9 kJ/mol). In this case, the orientation of the lactam group leads to a translation of the whole guest while the NH hydrogen bond acceptor is now the nucleobase G2540. Interestingly, the B ring in **23** does not insert into the A-site cleft. The same conformation and H-bond is adopted by our guest **24** (ΔΔ*E_b_* +25.8 kJ/mol). Candidate **25** superimposes well with the original position of linezolid itself. The pyrrole ring interacts via a strong H-bond (1.79 Å) with the G2540 phosphate group making this guest comparable to linezolid in terms of its remarkable relative binding affinity of +1.3 kJ/mol. A small negative relative binding energy is finally observed for guest **26**. The lowest energy minimum of the ribosomal complex with this guest shows that now the guest is bound upside down: the pyrrole NH group establishes an effective H-bond with the nucleobase U2620 located at the A-site entry border. The morpholine group is now inside the active site while the B ring is still occupying the A-site cleft making guest **26** a better binder than linezolid by −7.0 kJ/mol. Nevertheless this value is still above the −15 kJ/mol which was defined by us as a lower bound for the prediction of an effective binder after the analysis of Table 1.

The candidate with the most-promising affinity was identified finally by oxazolidinone **20** comprising a new binding mode and a relative binding energy of −12.9 kJ/mol. While the bicyclic system is causing the molecule to move outwards (as in the case of **27** and **28**), this time, the oxazolidinone substructure replaces the original B ring position interacting with the A-site cleft. The saturated portion of the bicyclic system does not clash against anything being faced to the protein exit tunnel. The B ring that moved outwards is now stacking-interacting almost face-to-face with the phenyl moiety of CCA-Phe. A strong hydrogen bond (1.81 Å; relaxed force constant of 0.33 N/cm) is observed between the side chain oxygen and the G2540 nucleobase NH_2_. A simple hydrogenation of the bicyclic double bond changes the relative binding affinity dramatically. Guest **21** is destabilized by nearly 70 kJ/mol in comparison with the guest **20**. The increased steric effect is once again rising the energy of the complex and pushing the ligand outside the binding site.

## Conclusion

The proposed combination of computational power and chemical intuition led to the prediction of several new and promising antibacterial lead agents for treatment of serious Gram-positive infections. Settled right between very fast, but often erratic, virtual screening techniques, on the one side, and alchemical free energy perturbation calculations on the other, our protocol speed (one day per guest on a single modern computing node) allows for a screening of tens of candidates per day in production mode. Our multistep strategy relies on the reproduction of 1) experimentally known ribosome–drug complex geometries, 2) experimentally known ribosome–drug complex affinities 3) the known enantioselectivity of linezolid and 4) the existence and strength of the pivotal hydrogen bond between linezolid’s side chain and the ribosome calculated by means of our proposed compliance constants protocol. The proposed strategy might lead to a robust discrimination between effective and ineffective linezolid-like drug candidates, based on a data set of several hundred small organic molecules within a month. The combination of chemical intuition and computational power, the rationalization of good and bad binders in terms of an atomistic resolution inherent in our in silico model, will itself lead to novel candidate suggestions, verifiable by the proposed ribosomal model.

## Supporting Information

File 1 Computational details.
