# Correction: Effective in silico prediction of new oxazolidinone antibiotics: force field simulations of the antibiotic–ribosome complex supervised by experiment and electronic structure methods

**DOI:** 10.3762/bjoc.12.59

**Published:** 2016-03-31

**Authors:** Jörg Grunenberg, Giuseppe Licari

**Affiliations:** 1 Institut für Organische Chemie, Hagenring 30, TU-Braunschweig, 38106 Braunschweig, Germany; 2 Physical Chemistry Department, Sciences II, University of Geneva, 30, Quai Ernest Ansermet, CH-1211 Geneva 4, Switzerland

**Keywords:** compliance constants, computational chemistry, drug design, molecular recognition, relaxed force constants

Our original publication contains an erratic number of predicted antibiotic structures in Scheme 2. With this Erratum we provide the corrected Scheme 2.

**Scheme 1:**
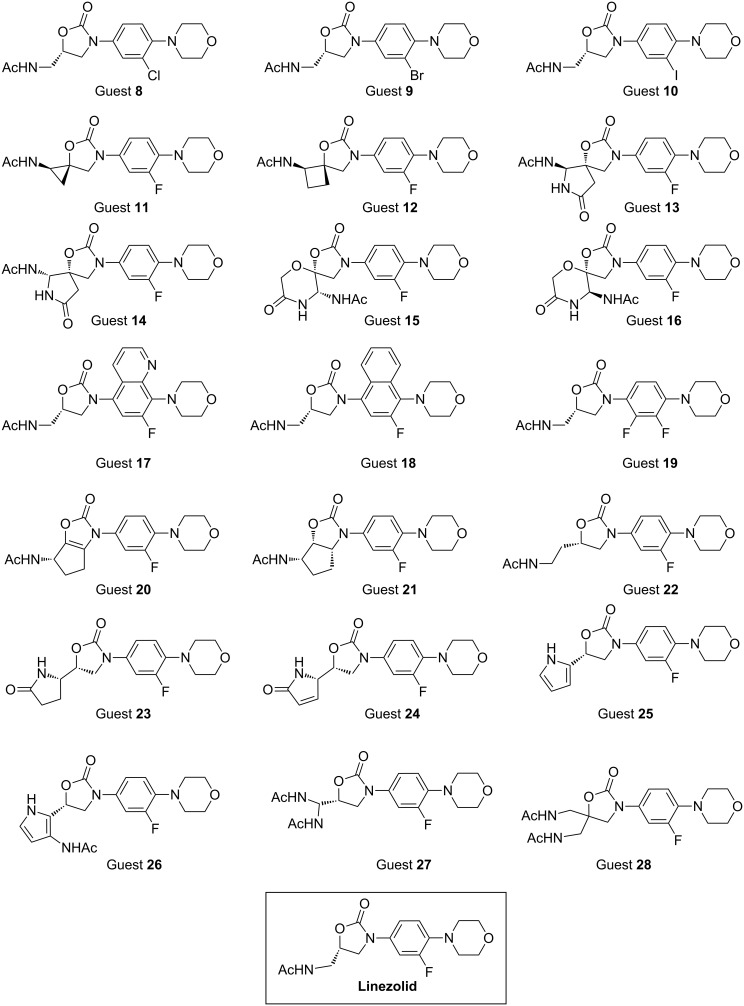
**Scheme 2 in the original article:** Predicted new linezolid-like candidates.

