# Supplementary material for: Effective in silico prediction of new oxazolidinone antibiotics: force field simulations of the antibiotic–ribosome complex supervised by experiment and electronic structure methods
Source: Beilstein J Org Chem. 2016 Mar 4;12:415–28. doi: 10.3762/bjoc.12.45 (PMC4902031; doi:10.3762/bjoc.12.45)
Supplement: File 1 — Computational details. [file Beilstein_J_Org_Chem-12-415-s001.pdf]

Supporting Information

for

Effective in silico prediction of new oxazolidinone antibiotics: force field simulations of the antibiotic–ribosome complex supervised by experiment and electronic structure methods

Jörg Grunenberg[*,1] and Giuseppe Licari[*,1,2]

Address: [1]Institut für Organische Chemie, Hagenring30, TU-Braunschweig, 38106 Braunschweig, Germany and [2]present address: Physical Chemistry Department, Sciences II, University of Geneva , 30, Quai Ernest Ansermet, CH-1211 Geneva 4, Switzerland

Email: Jörg Grunenberg - Joerg.Grunenberg@tu-bs.de; Giuseppe Licari - Giuseppe.Licari@unige.ch

*Corresponding author

Computational details

Molecular mechanics calculations were carried out using the MacroModel software package applying the AMBER* [1] and the OPLS_2005 [2] implementation as it is, including the description of the atomic charges for unknown molecules. All charges were taken from the FFs, even for linezolid and other guests. For Amber*, the charges were obtained by a fitting procedure to the electrostatic potential employing the RESP (restrained ESP-fit)[1] charge model at the HF/6-31G* level of theory. For the OPLS family of force fields, the charges have been obtained from a fitting process in order to reproduce organic liquids properties.[2] The OPLS_05 version is a Schrodinger optimized version of OPLS all atom force field, which provides a larger coverage of organic functionality. Additional charges have been fit at a high level of quantum theory.

Besides the explicit inclusion of several important water molecules, implicit solvent effects were taken into account using the GB/SA model [3,4]. All atomic charges were supplied by the individual force field. The cutoff for the long range interactions were set to 7.0 Å for van der Waals forces, 4.0 Å for the H-bonds and 12.0 Å for the electrostatic interactions. During the MM geometric optimizations, following each MC step, the PRCG (Polak-Ribiere Conjugate Gradient) [5] method was used. A gradient convergence of 0.05 was applied unless otherwise specified in the paper. Conformational scans were performed using the hybrid Monte Carlo/Low Frequency Mode (MCMM/LMCS) algorithm [6,7], which has already been used successfully for exploring the conformational space of biological active ligands into their active sites [8,9]. Search variables were defined automatically during the MC runs. Because of the still considerable complexity of our ribosomal model, not all internal degrees of freedom were included as scan parameters.

Nevertheless, during the subsequent optimizations the systems full flexibility was considered. The maximal molecular translation was set to 0.5 Å, while the torsion/translation ratio was set to 0.8.

We repeated our protocol calculating the various conformations of linezolid inside the ribosome, applying the OPLS-AA force field. Indeed both, the global minimum and the 10 lowest conformations are characterized by a side chain, which is oriented pseudo equatorial with respect to the plane of the molecule, instead of a bent conformation known from the bioactive solid-state conformation (see. Fig. S1) . The OPLS-AA force field seems to underrate the attractive long-range interactions between the amidic group and the oxazolidinone ring also in the bound state. We would like to emphasize that this inferiority of the OPLS-AA force field is not an overall phenomenon. It rather underlines the importance of a thorough force field evaluation for each survey. See. Figure. S1.

The energy window for saving structures was set to 40 kJ/mol. Redundant conformers were eliminated using the maximum atom deviation with a cutoff of 0.5 Å. An overall number of 2000 MC steps were carried out for the solvated linezolid and its analogues in our production runs. Preliminary control runs with more steps did not produce lower global minima. A similar argument was used to establish a maximum of 10000 MC steps for the ribo/guest complexes. Calculations at the DFT level of theory were performed with Gaussian09 program [10] applying the m062x functional [11] and a standard triple zeta basis set, 6-311g(d,p). The calculated energies are based on zero point energy corrected SCF energies. Solvation effects in our qiantum chemical calculations were considered through the default IEFPCM model [12]. Relaxed force constants were calculated for selected complexes applying our Compliance algorithm (version 3.0.2) [13].

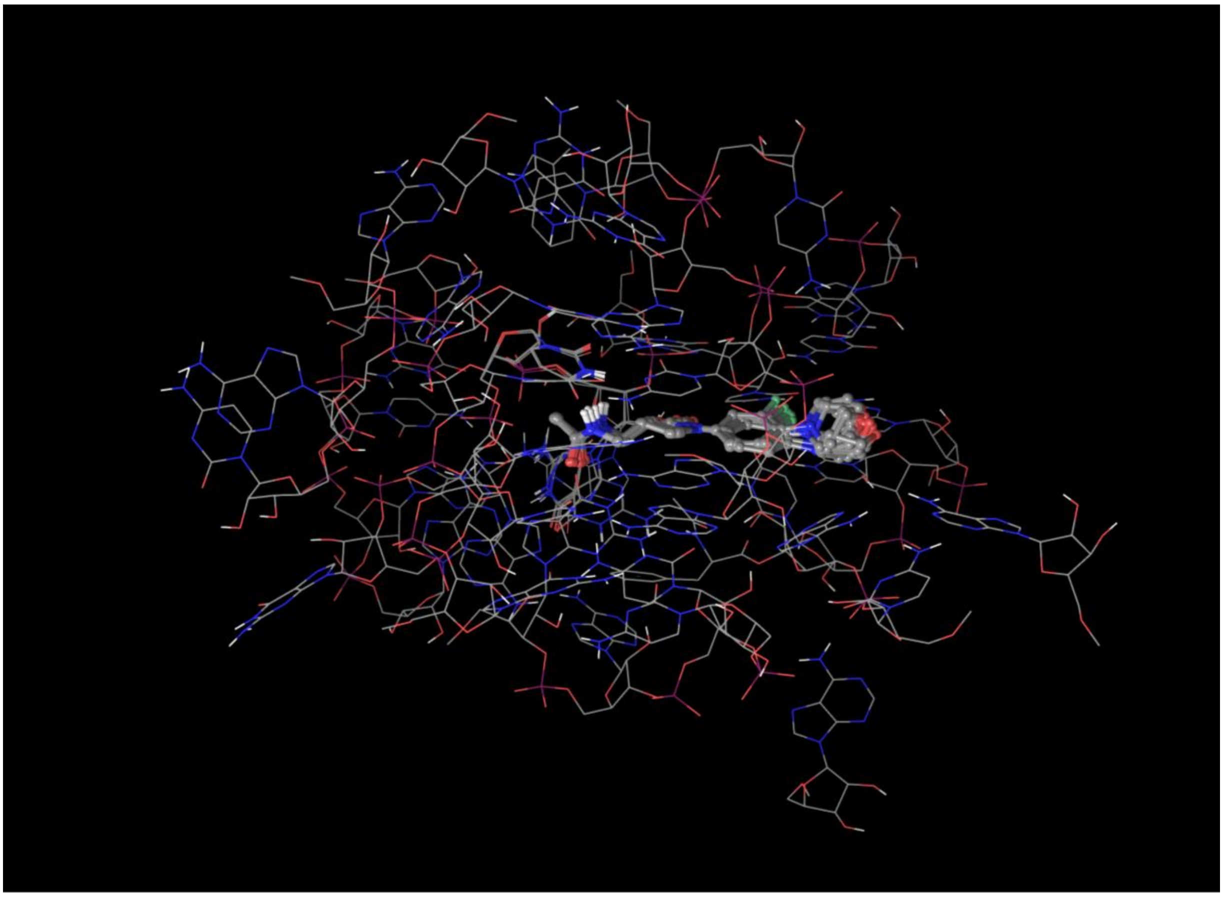

Figure S1: Superposition of 10 low energy minima of the ribosome/linezolid complex, computed at the OPLS-AA level of theory. Linezolid's side chain is oriented pseudo equatorial with respect to the plane of the molecule, instead of a bent conformation known from the bioactive solid-state conformation.

Literature

1. Cornell, W. D.; Cieplak, P.; Bayly,C. I. ; Gould, I. R.; Merz, K. M.; Ferguson, D. M.; Spellmeyer, D. C.; Fox, T.; Caldwell, J. W.; Kollman, P. A. *J. Am. Chem. Soc.,* **1995**, 5179-5197.

2. Jorgensen, W. L.; Maxwell, D. S.; Tirado-Rives, J. *J. Am. Chem. Soc.* **1996**, 118, 11225-11236.

3. Still, W. C.; Tempczyk, A.; Hawley, R. C.; Hendrickson, T. *J. Am. Chem. Soc.* **1990**, 112, 6127-6129.

4. Qiu, D.; Shenkin, P. S.; Hollinger, F. P.; Still, W. C. *J. Phys. Chem. A* **1997**, 101, 3005-3014.

5. Polak, E.; Ribiere, G. *Rev. Franc. Inform. Rech. Oper.* **1969**, 16, 35-43.

6. Kolossvary, I.; Guida, W. C. *J. Am. Chem. Soc.* **1996***,* 118, 5011-5019.

7. Kolossváry, I. & Guida, W. C. *J. Comput. Chem.* **1999**, 20, 1671-1684.

8. Chang, G.; Guida, W. C.; Still, W. C. *J. Am. Chem. Soc.* **1989**, 111, 4379-4386.

9. Saunders, M.; Houk, K. N.; Wu, Y. D.; Still, W. C.; Lipton, M.; Chang, G.; Guida, W. C. *J. Am. Chem. Soc.* **1990***,*112, 1419-1427 (1990).

10. Frisch, M. J.; Trucks, G. W.; Schlegel, H. B.; Scuseria, G. E.; Robb, M. A.; Cheeseman, J. R.; Scalmani, G.; Barone, V.; Mennucci, B.; Petersson, G. A.; Nakatsuji, H.; Caricato, M.; Li, X.; Hratchian, H. P.; Izmaylov, A. F.; Bloino, J.; Zheng, G.; Sonnenberg, J. L.; Hada, M.; Ehara, M.; Toyota, K.; Fukuda, R.; Hasegawa, J.; Ishida, M.; Nakajima, T.; Honda, Y.; Kitao, O.; Nakai, H.; Vreven, T.; Montgomery, J. A., Jr.; Peralta, J. E.; Ogliaro, F.; Bearpark, M.; Heyd, J. J.; Brothers, E.; Kudin, K. N.; Staroverov, V. N.; Kobayashi, R.; Normand, J.; Raghavachari, K.; Rendell, A.; Burant, J. C.; Iyengar, S. S.; Tomasi, J.; Cossi, M.; Rega, N.; Millam, J. M.; Klene, M.; Knox, J. E.; Cross, J. B.; Bakken, V.; Adamo, C.; Jaramillo, J.; Gomperts, R.; Stratmann, R. E.; Yazyev, O.; Austin, A. J.; Cammi, R.; Pomelli, C.; Ochterski, J. W.; Martin, R. L.; Morokuma, K.; Zakrzewski, V. G.; Voth, G. A.; Salvador, P.; Dannenberg, J. J.; Dapprich, S.; Daniels, A. D.; Farkas, Ö.; Foresman, J. B.; Ortiz, J. V.; Cioslowski, J.; Fox, D. J. Gaussian, Inc., Wallingford CT, 2009 (Gaussian, Inc., Wallingford CT, 2009).

11. Zhao, Y.; Truhlar, D. G. *Acc. Chem. Res.* **2008**, 41, 157-167.

12. Tomasi, J., Mennucci, B.; Cancès, E. *J. Mol. Struct.* **1999**, 464, 211-226.

13. Brandhorst, K.; Grunenberg, J. *J. Chem. Phys.* **2010**, 132, 184101-7.
